# *PAI-1* 4G/5G Polymorphism Contributes to Cancer Susceptibility: Evidence from Meta-Analysis

**DOI:** 10.1371/journal.pone.0056797

**Published:** 2013-02-20

**Authors:** Shangqian Wang, Qiang Cao, Xiaoxiang Wang, Bingjie Li, Min Tang, Wanqing Yuan, Jianzheng Fang, Jian Qian, Chao Qin, Wei Zhang

**Affiliations:** 1 Department of Urology, The First Affiliated Hospital of Nanjing Medical University, Nanjing, China; 2 Department of Urology, Yangzhou No.1 People’s Hospital, Yangzhou, China; 3 Division of Epidemiology, University of Texas Health Science Center, Houston, Texas, United States of America; 4 Department of Orthopedics, The Affiliated Hospital of Medical College, Qingdao University, Qingdao, China; University of Montreal, Canada

## Abstract

**Background:**

The plasminogen activator inhibitor-1 (*PAI-1*) is expressed in many cancer cell types and allows the modulation of cancer growth, invasion and angiogenesis. To date, studies investigated the association between a functional polymorphism in *PAI-1* (4G/5G) and risk of cancer have shown inclusive results.

**Methods:**

A meta-analysis based on 25 case-control studies was performed to address this issue. Odds ratios (OR) with corresponding 95% confidence intervals (CIs) were used to assess the association. The statistical heterogeneity across studies was examined with *I^2^* test.

**Results:**

Overall, a significant increased risk of cancer was associated with the *PAI-1* 4G/4G polymorphism for the allele contrast (4G vs. 5G: OR = 1.10, CI = 1.03–1.18, *I^2^* = 49.5%), the additive genetic model (4G/4G vs. 5G/5G: OR = 1.21, CI = 1.06–1.39, *I^2^* = 51.9%), the recessive genetic model (4G/4G vs. 4G/5G+5G/5G: OR = 1.11, CI = 1.04–1.18, *I^2^* = 20.8%). In the subgroup analysis by ethnicity, the results indicated that individuals with 4G/4G genotype had a significantly higher cancer risk among Caucasians (4G/4G vs. 5G/5G: OR = 1.31, 95%CI = 1.09–1.59, *I^2^* = 59.6%; 4G/4G vs. 4G/5G: OR = 1.12, 95%CI = 1.04–1.21, *I^2^* = 3.6%; recessive model: OR = 1.12, 95%CI = 1.05–1.21, *I^2^* = 25.3%).

**Conclusions:**

The results of the present meta-analysis support an association between the *PAI-1* 4G/5G polymorphism and increasing cancer risk, especially among Caucasians, and those with 4G allele have a high risk to develop colorectal cancer and endometrial cancer.

## Introduction

The urokinase plasminogen activator system is a serine protease family [1]. The included urokinase-type plasminogen activator (uPA)system provides the most substantial amount of activated plasminogen when tissues are being degraded and is involved in extracellular matrix (ECM) degradation [2], [3], hence it has been involved in numerous pathophysiological processes requiring the remodeling of basement membranes (BM) and ECM. Metastasis and invasion of malignant cancers require proteolytic degradation of the ECM, BM and infiltration of cancer cells into the surrounding tissues, the blood stream, or the lymphatic vessels. Studies revealing the uPA system, universal to all cancers, is associated with the process of cancer metastasis and progression by participating in the degradation and regeneration of the BM and ECM [4], [5], [6].

The plasminogen activator inhibitor-1 (*PAI-1*), a 52 kDa glyco-protein belong to the serine proteinase inhibitor super family, is a multifaceted proteolytic factor. It is the principal inhibitor of tissue and urinary plasminogen activators, and therefore constitutes an important regulatory protein in fibrinolysis [7], [8]. It is also involved in the regulation of cell adhesion, detachment and migration, playing an important role in cancer progression [9], [10], [11]. Indeed, *PAI-1* is expressed in many types of cancer cell and allows the modulation of cancer growth, invasion and angiogenesis in a dose-dependent manner [12].

Genetic polymorphisms in the *PAI-1* gene seem to contribute to the level of *PAI-1* biosynthesis [13]. A single nucleotide insertion/deletion (4G/5G) polymorphism located at 675 base-pair (bp) upstream of the transcriptional start site in the *PAI-1* promoter, is the most frequently studied variant because of its possible involvement in the regulation of *PAI-1* transcription [14], [15], [16]. Based on the investigation by CDC (Centers for Disease Control and Prevention), the 4G/5G allele frequencies range in various populations from 26.7/73.3% to 52.5/47.5%,respectively. (http://www.cdc.gov/genomics/population/genvar/frequencies/SERPINE1.htm). The distribution of 4G/5G allele frequencies has been shown in Table S1.

Homozygosity of the 4G allele is considered to be a risk factor for developing deep vein thrombosis, myocardial infarction and high rate of miscarriage during pregnancy [17], [18], [19]. Many molecular epidemiological studies have been conducted to investigate the association between 4G/5G polymorphism and cancer risk in humans [20]–[43]. However, the results from these studies are to some extent divergent, but nevertheless intriguing, which may be owe to limitations in individual studies. To address this issue, we performed a meta-analysis with subgroup analysis from all eligible studies, to obtain a more precise estimation of the relationship between *PAI-1* 4G/5G polymorphism and cancer risk.

**Table 1:** Characteristics of studies included in the meta-analysis.

First author	Ethnicity	Country	Cancer	Genotyping	Source ofControls	Sample size		HWE
						case	control	
Turkmen 1997	Caucasian	Germany	Ovarian cancer	PCR-RFLP	HB	22	23	Y
Smolarz 1999	Caucasian	Poland	Breast cancer	Allele-specific PCR	HB	37	53	Y
Blasiak 2000	Caucasian	Poland	Breast cancer	Allele-specific PCR	HB	100	106	Y
Loktionov 2003	Caucasian	UK	Colorectal	PCR-RFLP	HB	206	355	Y
Castello 2006	Caucasian	Spain	Breast cancer	Allele-specific PCR	HB	104	104	Y
Eroglu 2006	Caucasian	Turkey	Breast cancer	PCR-RFLP	HB	34	90	Y
Sternlicht 2006	Caucasian	UK	Breast cancer	PCR-RFLP	PB	2539	1832	Y
Eroglu 2007	Caucasian	Turkey	Others	PCR-RFLP	HB	125	180	Y
Forsti 2007	Caucasian	Sweden	Colorectal cancer	Taqman	PB	304	581	Y
Jorgenson 2007	Mixed	USA	Prostate cancer	PCR-RFLP	PB	638	478	Y
Minisini 2007	Caucasian	Italy	Breast cancer	Allele-specific PCR	HB	193	142	Y
Woo 2007	Asian	Korea	Colorectal cancer	PCR-RFLP	HB	185	304	Y
Lei 2008	Caucasian	Sweden	Breast cancer	Taqman	PB	956	943	Y
Bentov 2009	Mixed	Canada	Ovarian cancer	MassARRAY	PB	772	889	Y
Palmirotta 2009	Caucasian	Italy	Breast cancer	PCR-RFLP	HB	99	50	Y
Vairaktaris 2009	Caucasian	Greece Germany	Oral cancer	PCR-RFLP	HB	104	106	Y
Ju 2010	Asian	Korea	Gastric cancer	MassARRAY	PB	252	406	Y
Weng 2010	Asian	Taiwan	Hepatocellular cancer	PCR-RFLP	HB	102	344	Y
Gilabert-Estelles 2011	Caucasian	Spain	Endometrial cancer	Allele-specific PCR	HB	212	211	Y
Su 2011	Asian	Taiwan	Endometrial	PCR-RFLP	HB	134	302	Y
Vossen 2011	Caucasian	Germany	Colon cancer	Taqman	PB	1059	1799	Y
Vossen 2011	Caucasian	Germany	Rectal cancer	Taqman	PB	672	1799	Y
Weng 2011	Asian	Taiwan	Oral cancer	PCR-RFLP	HB	253	344	Y
Onur 2012	Caucasian	Turkey	Others	Two parallel PCR	HB	28	50	Y
Tee 2012	Asian	Taiwan	Cervical cancer	PCR-RFLP	HB	75	336	Y

HB, hospital based; PB, population based; HWE,Hardy–Weinberg equilibrium.

## Materials and Methods

### Identification and Eligibility of Relevant Studies

All case-control studies on the association between *PAI-1* polymorphisms and cancer risk published up to July 31, 2012 were identified through comprehensive searches using the PubMed and EMBASE database with the following terms and keywords: “plasminogen activator inhibitor-1″, “*PAI-1”* and “polymorphism”, “variation”, “mutation” and in combination with “cancer”, “tumor” and “carcinoma”. The search was limited to human studies and English language papers.

### Inclusion Criteria

For inclusion in the meta-analysis, the identified articles have to meet the following criteria: (a) there is information on the evaluation of the *PAI-1* 4G/5G polymorphism and cancer risk, (b) using a case-control design, and (c) containing complete information about all genotype frequency. The exclusion criteria are as follows: (a) not for cancer research, (b) review articles, (c) reports without usable data and (d) duplicate publications.

### Data Extraction

Information was carefully extracted from all the eligible publications independently by two researchers (SQ Wang and Q Cao) according to the inclusion criteria listed above. For conflicting evaluation, a consensus was reached by discussion. The following information was extracted from each included study using a standardized data collection protocol (File S1): the first author’s name, the year of publication, ethnicity, country of origin, cancer type, genotyping method and source of control groups (population- or hospital-based controls) and deviation from Hardy-Weinberg Equilibrium (HWE) of the control group. Different ethnic descents were categorized as African, Asian, European, or Mixed (composed of different ethnic groups). Meanwhile, different case-control groups in one study were considered as independent studies.

### Statistical Methods

The strength of the association between the *PAI-1* 4G/5G polymorphism and cancer risk was measured by odds ratios (ORs) with corresponding 95% confidence intervals (CIs). The percentage weight determined by the precision of its estimate of effect and, in the statistical software in STATA and SAS, is equal to the inverse of the variance. The risks (ORs) of cancer associated with the *PAI-1* 4G/5G polymorphism were estimated for each study. In our study, the 5G allele was considered the reference genotype. The pooled ORs were performed for additive genetic model (4G/4G vs. 5G/5G and 4G/4G vs. 4G/5G), dominant model (4G/4G +4G/5G vs. 5G/5G) and recessive model (4G/4G vs. 4G/5G +5G/5G), respectively. Stratified analyses were also performed by cancer types (if one cancer type contained less than two individual studies, it was classified as other cancers group), ethnicity, source of controls and sample size (subjects ≥500 in both case and control groups or not). In consideration of the possibility of heterogeneity across the studies, a statistical test for heterogeneity was performed by a *I^2^* test. *I^2^*value and its 95%/97.5CI were both calculated and shown in Table S2. A *I^2^* smaller than 31% indicates lack of heterogeneity among the studies, and then the fixed-effects model (the Mantel-Haenszel method) was used to calculate the summary OR estimate of each study. Otherwise, the random effects model (DerSimonian and Laird method) was used. For each study, we examined whether the genotype distribution of controls was consistent with HWE using the *χ2* test. One-way sensitivity analysis was performed to assess the stability of the results, namely, a single study in the meta-analysis was deleted each time to reflect the influence of the individual data set to the pooled OR. An estimate of potential publication bias was carried out by the funnel plot, in which the standard error of log (OR) of each study was plotted against its log (OR). An asymmetric plot suggests a possible publication bias. Funnel plot asymmetry was assessed by the method of Egger’s linear regression test, a linear regression approach to measure funnel plot asymmetry on the natural logarithm scale of the OR. All statistical analyses were performed with the Stata software (version 12.1; StataCorp LP, College Station, TX, USA) and SAS software (Version 9.2; SAS Institute, Cary, NC, USA),using two-sided *P*-values.

## Results

### Characteristics of Studies

**Figure 1:**
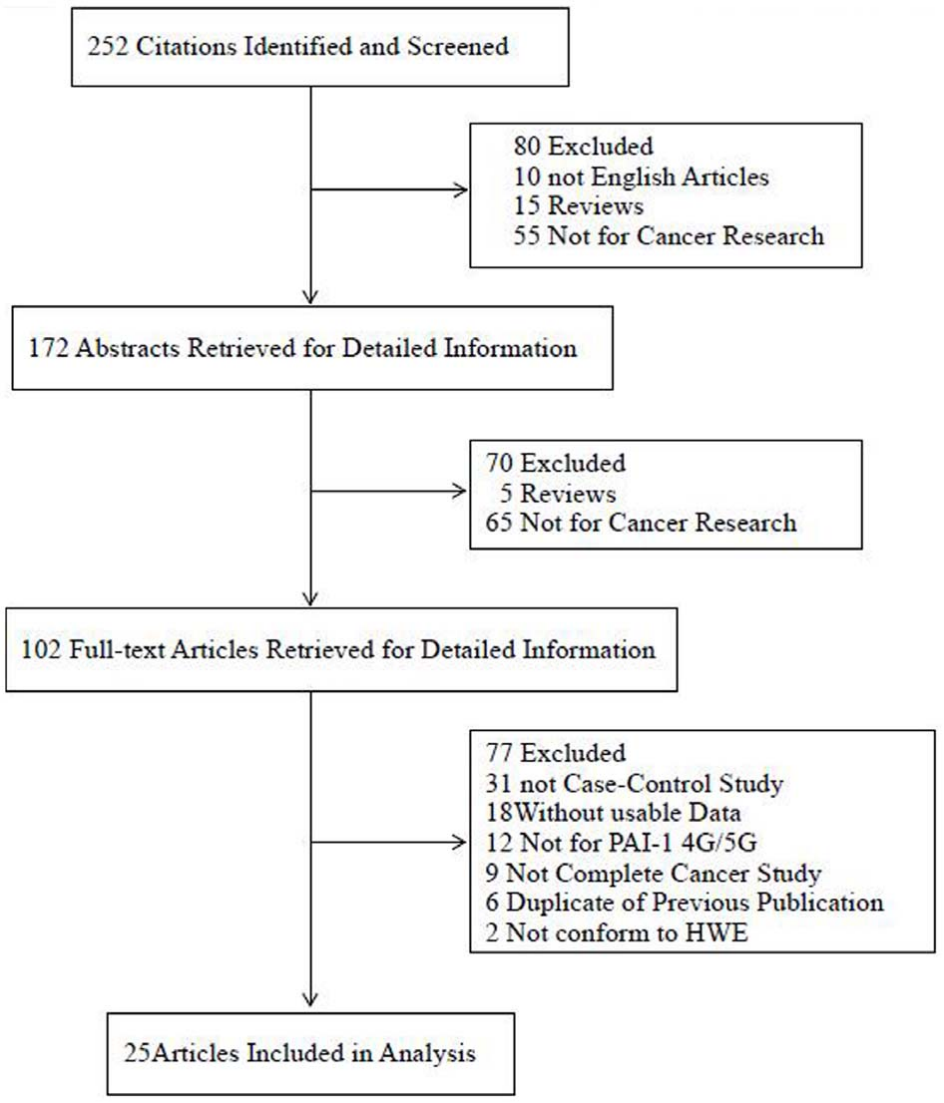
Studies identified with criteria for inclusion and exclusion.

There were 25 studies retrieved on the basis of the search criteria (Fig. 1). Totally, 9,205 cases and 11,827 controls were included in the meta-analysis. Study characteristics were summarized in Table S1. Among the 25 case–control studies, there were 17 studies of Caucasians, 6 studies of Asians, and 2 studies of mixed descendents. There were 8 breast cancer studies, 5 colorectal cancer studies, 2 ovarian cancer studies, 2 endometrial cancer studies, 2 oral cancer studies, and the others were categorized into the “other cancer” group. Cancers were confirmed histologically or pathologically in most studies. Controls were mainly matched on sex and age, of which 17 were hospital based [21], [22], [23], [24], [29], [30], [31], [32], [33], [35], [36], [37], [38], [40], [41], [42], [43], 8 were population based [20], [25], [26], [27], [28], [34], [39]. Furthermore, 10 studies were conducted with subjects >500 in both case and control groups [20], [25], [26], [27], [28], [29], [34], [39], [40]. Diverse genotyping methods were used, including PCR–RFLP, TaqMan, allele-specific PCR, MassARRAY, and two parallel PCR. The distribution of genotypes in the controls of all studies was consistent with HWE.

### Quantitative Synthesis

**Figure 2:**
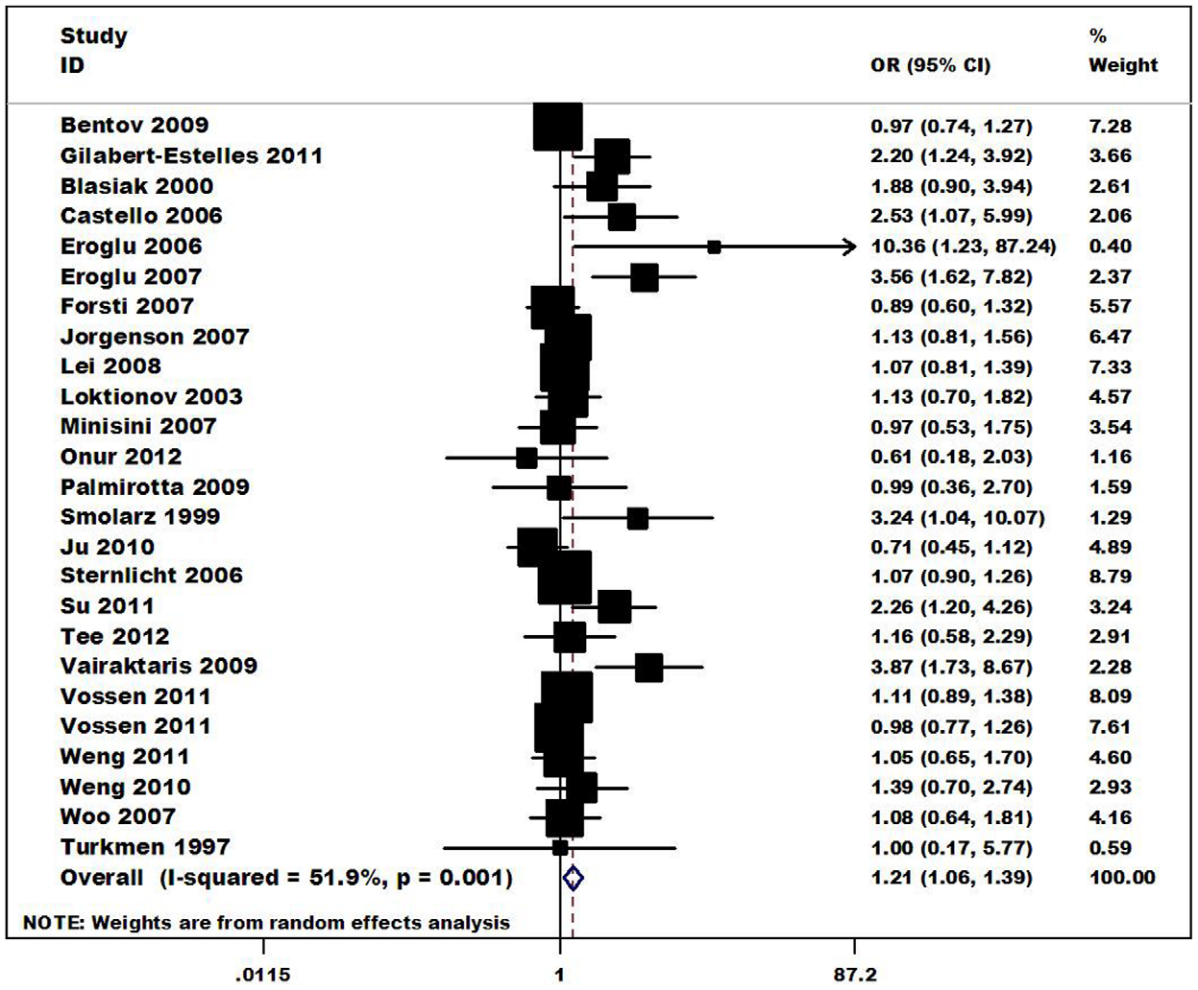
Forest plot of cancer risk associated with the *PAI-1* 4G/5G polymorphism (4G/4G vs. 5G/5G). The squares and horizontal lines correspond to the study-specific OR and 95% CI. The area of the squares reflects the weight (inverse of the variance). The diamond represents the summary OR and 95% CI.

**Figure 3:**
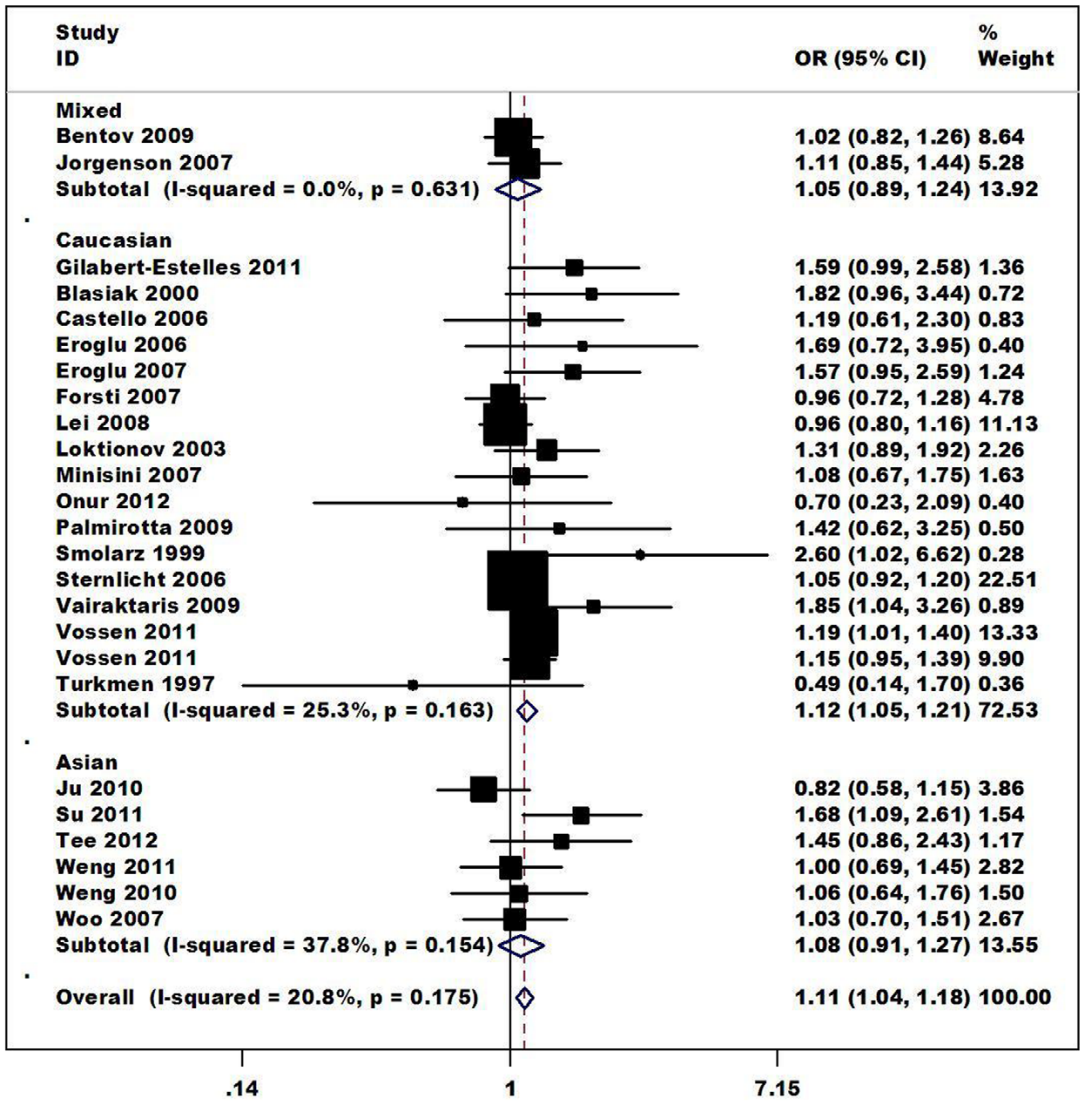
Forest plot of cancer risk associated with the *PAI-1* 4G/5G polymorphism by Ethnicity (recessive model). The squares and horizontal lines correspond to the study-specific OR and 95% CI. The area of the squares reflects the weight (inverse of the variance). The diamond represents the summary OR and 95% CI.

The relationship between the 4G/5G polymorphism in *PAI-1* and the risk of different kinds of cancer are summarized in Table 2. Overall, a significantly increased risk of cancer was is associated with the *PAI-1* 4G polymorphism for the allele contrast (4G vs. 5G: OR = 1.10, CI = 1.03–1.18, *I^2^* = 49.5%), the additive genetic model (4G/4G vs. 5G/5G: OR = 1.21 CI = 1.06–1.39, (Fig. 2); 4G/4G vs. 4G/5G: OR = 1.10 CI = 1.03–1.18), the recessive genetic model (4G/4G vs. 4G/5G+5G/5G OR = 1.11 CI = 1.04–1.18). In the subgroup analysis by ethnicity, the results indicated that individuals with 4G/4G genotype had a significantly higher cancer risks among Caucasians (4G/4G vs. 5G/5G: OR = 1.31, 95%CI = 1.09–1.59; 4G/4G vs. 4G/5G: OR = 1.12, 95%CI = 1.04–1.21; recessive model: OR = 1.12, 95%CI = 1.05–1.21), (Fig. 3). When restricting the analysis to the source of controls, significant associations were found in Hospital-based source (4G/4G vs. 5G/5G: OR = 1.59, 95%CI = 1.24–2.05; 4G/4G vs. 4G/5G: OR = 1.22, 95%CI = 1.07–1.40; dominant model: OR = 1.38, 95%CI = 1.10–1.74; recessive model: OR = 1.30, 95%CI = 1.14–1.48). In the stratified analysis by cancer types, significant associations were found for Endometrial cancer (4G/4G vs. 5G/5G: OR = 2.23, 95%CI = 1.45–3.42; 4G/4G vs. 4G/5G: OR = 1.45, 95%CI = 1.04–2.04; dominant model: OR = 1.74, 95%CI = 1.23–2.47; recessive model: OR = 1.64, 95%CI = 1.19–2.27), Colorectal cancer (4G/4G vs. 4G/5G OR = 1.19, 95%CI = 1.06–1.33; recessive model: OR = 1.14, 95%CI = 1.03–1.27). In the stratified analysis by sample size (both cases and controls), significant associations were found for <500 (4G/4G vs. 5G/5G: OR = 1.73, 95%CI = 1.31–2.31; 4G/4G vs. 4G/5G: OR = 1.24, 95%CI = 1.06–1.45; dominant model: OR = 1.49, 95%CI = 1.14–1.93; recessive model: OR = 1.36, 95%CI = 1.17–1.57).

**Table 2:** Stratification analyses of the *PAI-1* 4G/5G polymorphism on cancer.

Variables	Sample size			4Gvs5G		4G/4Gvs5G/5G		4G/4Gvs4G/5G		4G/4Gvs4G/5G+5G/5G	
	N^a^	case	control	OR(95% CI)	*I^2^(%)*	OR(95% CI)	*I^2^(%)*	OR(95% CI)	*I^2^(%)*	OR(95% CI)	*I^2^(%)*
**Total**	25	9205	11827	**1.10(1.03–1.18)**	49.5^c^	**1.21(1.06–1.39)**	51.9^c^	**1.10(1.03–1.18)**	0^c^	**1.11(1.04–1.18)**	20.8^c^
**Tumor type**											
Breast cancer	8	4062	3320	1.14(1.00–1.29)	48.3	1.30(0.99–1.70)	48.8	1.05(0.94–1.16)	6	1.07(0.97–1.18)	22.8
Colorectal cancer	5	2426	4838	1.03(0.96–1.11)	0	1.04(0.90–1.19)	0	**1.19(1.06–1.33)**	0	**1.14(1.03–1.27)**	0
Ovarian cancer	2	794	912	0.98(0.86–1.13)	0	0.97(0.74–1.27)	0	1.01(0.81–1.26)	55.1	1.00(0.81–1.23)	22.9
Endometrial cancer	2	346	513	**1.45(1.19–1.77)**	0	**2.23(1.45–3.42)**	0	**1.45(1.04–2.04)**	0	**1.64(1.19–2.27)**	0
Oral cancer	2	357	450	1.39(0.73–2.63)	87.3	1.94(0.54–6.91)	86.5	1.07(0.77–1.49)	0	1.20(0.88–1.64)	67.7
Others	6	1220	1794	1.08(0.90–1.30)	57.8	1.18(0.79–1.78)	63.2	1.07(0.89–1.28)	0	1.08(0.91–1.28)	24.6
**Ethnicity**											
Caucasian	17	6794	8424	**1.14(1.04–1.25)**	56.8	**1.31(1.09–1.59)**	59.6	**1.12(1.04–1.21)**	3.6	**1.12(1.05–1.21)**	25.3
Asian	6	1001	2036	1.07(0.92–1.25)	45.9	1.14(0.84–1.56)	44.8	1.07(0.90–1.28)	17.3	1.08(0.91–1.27)	37.8
Mixed	2	1410	1367	1.02(0.92–1.13)	0	1.03(0.84–1.27)	0	1.06(0.89.1.27)	0	1.05(0.89–1.24)	0
**Control source**											
Hospital based	17	2013	3100	**1.25(1.11–1.40)**	43	**1.59(1.24–2.05)**	48.1	**1.22(1.07–1.40)**	0	**1.30(1.14–1.48)**	0
Population based	8	7192	8727	1.02(0.97–1.07)	0	1.03(0.94–1.13)	0	1.07(0.99–1.15)	8.4	1.06(0.99–1.13)	0
**Sample size(both cases and controls)**											
<500	15	1554	2401	**1.30(1.14–1.48)**	40	**1.73(1.31–2.31)**	46.5	**1.24(1.06–1.45)**	0	**1.36(1.17–1.57)**	0
≥500^d^	10	7651	9426	1.02(0.98–1.07)	0	1.03(0.94–1.13)	0	**1.08(1.00–1.16)** e	5	1.06(0.99–1.14)	0

a Number of studies.

***I*** **^2^**The value of heterogeneity test.

c Fix-effects model was used when ***I^2^*** value for heterogeneity test <31%; otherwise, random-effects model was used.

d Stratified according to subjects ≥500 in both case and control groups or not.

e The exact value is 1.077(1.002–1.156).

### Test for Heterogeneity

There was significant heterogeneity for allele contrast (4G vs. 5G: *I^2^* = 49.5%), homozygote comparison (4G/4G vs. 5G/5G: *I^2^* = 51.9*%*), heterozygote comparison (4G/5G vs. 5G/5G : *I^2^* = 48.7%), dominant model comparison (4G/4G+4G/5G vs. 5G/5G: *I^2^* = 53.9%), recessive model comparison (4G/4G vs. 4G/5G+5G/5G: *I^2^* = 20.8%). Then, we used a meta-regression analysis to explore the source of heterogeneity for homozygote comparison (4G/4G vs. 5G/5G) by Ethnicity, cancer types, source of controls and sample size. We found that the sample size (τ^2^ = 0, *P* = 0.001) contributed to substantial altered heterogeneity, which could account for 100% source of heterogeneity. Also, control source (τ^2^ = 0, *P* = 0.005) contributed to 100% source of heterogeneity. However, we did not find cancer types (τ^2^ = 0.074, *P* = 0.615), or ethnicity (τ^2^ = 0.075, *P* = 0.947) contributed to source of heterogeneity.

### Sensitivity Analysis

The sensitivity analysis was conducted by leaving out certain studies, such as the study that did not conform to HWE. The omission of individual studies did not materially alter the results, although on some occasions, the *I^2^* value for heterogeneity was reduced. The sensitivity analysis thus confirmed that the results of this meta-analysis were statistically robust. This procedure proved that our results were reliable and stable. Furthermore, when excluding the studies that were not in HWE, the estimated pool OR still did not change at all.

### Publication Bias

**Figure 4:**
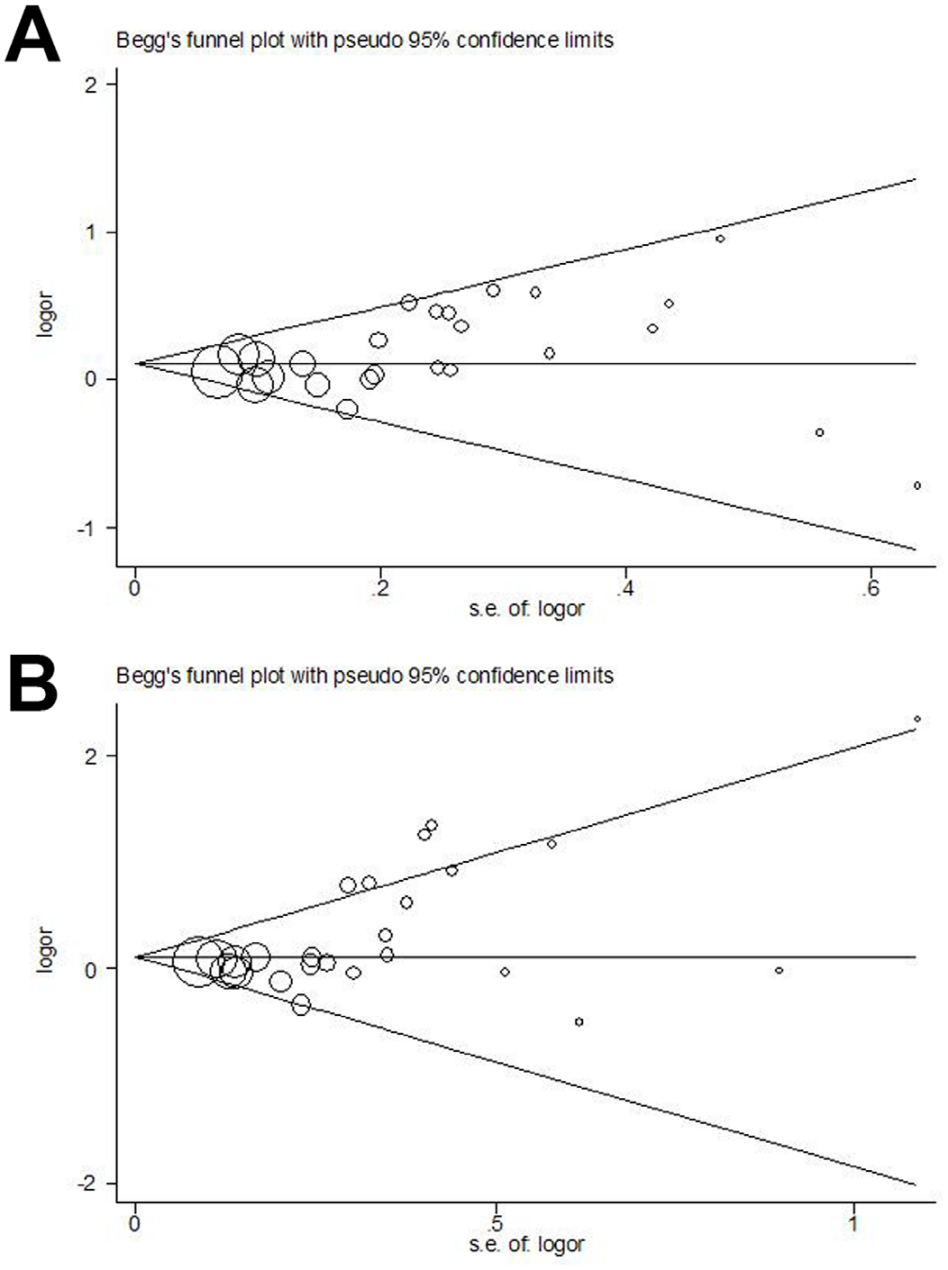
Begg’s funnel plot of publication bias test. (A) 4G/4G vs. 4G/5G+5G/5G. (B) 4G/4G vs. 5G/5G. Each point represents a separate study for the indicated association. Log(OR), natural logarithm of OR. Horizontal line, mean effect size.

Begg’s funnel plot and Egger’s test were performed to assess the publication bias of literatures. As shown in the Fig. 4, the shapes of the funnel plots seems symmetrical in the recessive genetic model (4G/4G vs. 4G/5G+5G/5G), but not for homozygote model(4G/4G vs. 5G5G). Thus, the Egger’s test was used to provide statistical evidence of funnel plot symmetry. For recessive genetic model, the results did not show any evidence of publication bias (t = 1.96, *P* = 0.097 for 4G/4G vs. 4G/5G+5G/5G). However, the homozygote model showed significant publication bias (t = 2.99 and *P* = 0.014). To adjust for this bias, a trim-and-fill method developed by Duval and Tweedie [44] was used to both identify and correct for funnel plot asymmetry arising from publication bias.We trimmed off the asymmetric outlying part of the funnel after estimating how many studies were in the asymmetric part with the help of Stata software. The result showed only one study should be trimmed after three times of iterations. We then estimated the ture center of the funnel and then replaced that trimmed study and its missing counterpart around the center. The final estimate of the true mean, and also its 95%CI, were then based on the filled funnel plot. The OR estimates and 95%CI in fixed-effect model before and after trim-and-fill were 1.119, (1.032–1.213) and 1.115, (1.029–1.209). Also, for random-effect model, the results were 1.214, (1.057–1.394) and 1.204, (1.049–1.392). Meta-analysis with or without the trim-and-fill method did not draw different conclusions, indicating that our results were statistically robust.

## Discussion

The present meta-analysis, including 9,205 cases and 11,827 controls from 25 case-control studies, explored the association between the *PAI-1* 4G/5G polymorphism and cancer risk. Our results indicated that the variant 4G/4G genotype was associated with an increased risk of cancers, especially of colorectal cancer and endometrial cancer.

In recent years, many studies have been conducted to investigate the associations between the *PAI-1* 4G/5G polymorphisms and disease risk across different countries. The results remain inconclusive. A new manuscript in Blood by Huang et al. [45] just published validated the role of the 4G/5G polymorphisms in circulating *PAI-1* levels using GWAS data. However, they revealed no association between *PAI-1* 4G/5G and type 2 diabetes (T2D) and coronary artery disease (CAD), despite enormous sample sizes.

Many researchers investigated the relationship between *PAI-1* blood concentrations and diseases risk. Palmirotta et al. [32] reported plasma *PAI-1* levels in breast cancer patients were approximately two fold higher than those observed in control subjects and were strongly dependent on cancer size, suggesting that cancer-related factors might be responsible for *PAI-1* up-regulation. However, the exact concentration of *PAI-1* in these breast cancer patients blood stayed 27.2 ng/ml (16.5–35.0), and Huang et al. [45] revealed cumulative effect of all common alleles explained extremely low blood levels of *PAI-1* in their GWAS data. How would such a seemingly small influence of genotype on *PAI-1* levels be expected to modify cancer risk? Given the important roles of *PAI-1* in multiple biological functions, such as regulation of cell adhesion, detachment and migration, it is biologically plausible that the *PAI-1* 4G/5G polymorphism may modulate the risk of cancers.Functional studies on this polymorphism have shown that the 4G allele binds only an activator, while the 5G allele binds a repressor as well as an activator, therefore results in reduced transcription of *PAI-1* [46]. It suggests that the 4G allele is associated with reduced inhibition of the plasminogen activators and, consequently, increased plasminogen conversion to plasmin, increased activation of MMPs and decreased adhesive strength of cells for their substratum [17], [18], [46]. Consistent with these observations, our meta-analysis showed that individuals carrying 4G/4G genotype were associated with a higher cancer risk than subjects carrying at least one 5G allele.

In addition, our results showed that the 4G allele may be a risk factor for colorectal cancer and endometrial cancer but not for breast cancer, ovarian cancer, oral cancer, or hepatocellular cancer. One factor that would contribute to the discrepancy among different studies is that this polymorphism might play a different role in different cancer sites. However, even at the same cancer site, considering the possible small effect size of this genetic polymorphism to cancer risk and the relatively small sample size in some studies, the discrepancy will become apparent since some of these studies may be underpowered to detect a small but real association. For endometrial cancer, there were only two studies included in the analysis with limited sample sizes, therefore, the results should be interpreted with caution.

In the subgroup analysis by ethnicity, an increased risk in 4G carriers was found among Caucasians but not Asians or Mixed. One explanation for this result may be that the studies using Mixed ethnicity participants enrolled them from various countries with diverse cultural, environmental and genetic characteristics. It is expected that these factors affected the synthesis results. On the other hand, the sample size and numbers of studies in Asian group were not adequate to evaluate the association. Other factors such as selection bias and different matching criteria may also play a role.

The genetic models were summarised in Table 2 including allele contrast model, homozygote model, heterozygote model and recessive model. Because of the strong heteogeneity in allele contrast and homozygote model, though, the results of these two shows significantly different, we do not suggest any one of these two as the best-fit model to represent the whole genetic models. There is a relatively low heterogeity (*I^2^* = 20.8%) in recessive model, the OR value and the confidence interval shows significantly different. As a result, the recessive model might be the best-fit model in this meta-analysis to reflect the whole results.

Furthermore, despite the overall robust statistical evidence generated through this analysis, some methodological limitations have been identified. Firstly, the relatively high heterogeneity and small sample size are the major defect in this meta-analysis. In the subgroup analyses by ethnicity and cancer type, the sample size of studies among Asians and among several cancer types is small and limited. As a result, the sample size accounted for most of the source of heterogeneity.Also, lacking the original data of the reviewed studies limites our further evaluation of potential interactions, because the interactions among gene-gene, gene-environment and even different polymorphic locis of the same gene may modulate cancer risk. Furthermore, the significant difference of results for hospital based control source should be interpreted in cautious. Accordingly, it is required that more studies be conducted to provide a more definitive conclusion that comprehensively explores the relationship between the *PAI-1* 4G/5G polymorphism and risk of cancer in the overall population.

### Conclusions

In conclusion, the evidence of the results from the present meta-analysis support an association between the *PAI-1* 4G/5G polymorphism and increasing cancer risk, especially among Caucasians, and those with colorectal cancer and endometrial cancer or cancers identified in the other cancers group, though significant heterogeneity from included studies existed. To advance an understanding of this relationship, the following recommendations have been made: (1) Large studies using standardized unbiased methods, enrolling precisely defined cancer patients and well matched controls, with more detailed individual data is needed. (2) Studies conducted with ethnic groups other than Caucasians are required to gain a more comprehensive and generalizable conclusion. (3) More and larger studies, especially studies stratified for gene-environmental interaction, should be performed to clarify the possible roles of the *PAI-1* 4G/5G polymorphisms in the etiology of cancer.

## Supporting Information

Table S1 **The genetype frequencies on each studies.** A generalized distribution of genetype frequencies on each included studies are listed.
(DOC)

Table S2 **Stratification analyses of the** ***I^2^*** **and 95%/97.5% confidence interval.** If *I^2^* = 0, the one-sided 97.5% CI is presented. Otherwise, a two-sided 95% CI is performed.
(DOC)

File S1 **PRISMA 2009 Checklist.**
(DOC)
