# Supplementary material for: PAI-1 4G/5G Polymorphism Contributes to Cancer Susceptibility: Evidence from Meta-Analysis
Source: PLoS One. 2013 Feb 20;8(2):e56797. doi: 10.1371/journal.pone.0056797 (PMC3577655; doi:10.1371/journal.pone.0056797)
Supplement: Table S1 — The genetype frequencies on each studies. A generalized distribution of genetype frequencies on each included studies are listed. (DOC) [file pone.0056797.s001.doc]

**Table S1. The genetype frequencies on each studies**

| **First author** | **Case** | | | | |  | **Control** | | | | |
| --- | --- | --- | --- | --- | --- | --- | --- | --- | --- | --- | --- |
|  | **4G4G** | **4G5G** | **5G5G** | **4G4G4G5G** | **4G5G5G5G** |  | **4G4G** | **4G5G** | **5G5G** | **4G4G4G5G** | **4G5G5G5G** |
| Bentov 2009 | 0.29 | 0.48 | 0.23 | 0.77 | 0.71 |  | 0.29 | 0.49 | 0.22 | 0.78 | 0.71 |
| Gilabert-Estelles 2011 | 0.24 | 0.56 | 0.2 | 0.8 | 0.76 |  | 0.17 | 0.53 | 0.31 | 0.69 | 0.83 |
| Blasiak 2000 | 0.31 | 0.4 | 0.29 | 0.71 | 0.69 |  | 0.2 | 0.45 | 0.35 | 0.65 | 0.8 |
| Castello 2006 | 0.23 | 0.63 | 0.13 | 0.87 | 0.77 |  | 0.2 | 0.5 | 0.3 | 0.7 | 0.8 |
| Eroglu 2006 | 0.35 | 0.62 | 0.03 | 0.97 | 0.65 |  | 0.24 | 0.54 | 0.21 | 0.79 | 0.76 |
| Eroglu 2007 | 0.34 | 0.57 | 0.09 | 0.91 | 0.66 |  | 0.25 | 0.52 | 0.23 | 0.77 | 0.75 |
| Forsti 2007 | 0.35 | 0.45 | 0.2 | 0.8 | 0.65 |  | 0.36 | 0.46 | 0.18 | 0.82 | 0.64 |
| Jorgenson 2007 | 0.28 | 0.47 | 0.25 | 0.75 | 0.72 |  | 0.26 | 0.48 | 0.26 | 0.74 | 0.74 |
| Lei 2008 | 0.34 | 0.5 | 0.16 | 0.84 | 0.66 |  | 0.35 | 0.48 | 0.17 | 0.83 | 0.65 |
| Loktionov 2003 | 0.29 | 0.46 | 0.25 | 0.75 | 0.71 |  | 0.24 | 0.53 | 0.23 | 0.77 | 0.76 |
| Minisini 2007 | 0.29 | 0.44 | 0.27 | 0.73 | 0.71 |  | 0.27 | 0.48 | 0.25 | 0.75 | 0.73 |
| Onur 2012 | 0.21 | 0.36 | 0.43 | 0.57 | 0.79 |  | 0.28 | 0.38 | 0.34 | 0.66 | 0.72 |
| Palmirotta 2009 | 0.26 | 0.44 | 0.29 | 0.71 | 0.74 |  | 0.2 | 0.58 | 0.22 | 0.78 | 0.8 |
| Smolarz 1999 | 0.41 | 0.38 | 0.22 | 0.78 | 0.59 |  | 0.21 | 0.43 | 0.36 | 0.64 | 0.79 |
| Ju 2010 | 0.31 | 0.48 | 0.21 | 0.79 | 0.69 |  | 0.35 | 0.48 | 0.17 | 0.83 | 0.65 |
| Sternlicht 2006 | 0.31 | 0.48 | 0.2 | 0.8 | 0.69 |  | 0.3 | 0.49 | 0.21 | 0.79 | 0.7 |
| Su 2011 | 0.37 | 0.5 | 0.13 | 0.87 | 0.63 |  | 0.25 | 0.53 | 0.21 | 0.79 | 0.75 |
| Tee 2012 | 0.39 | 0.4 | 0.21 | 0.79 | 0.61 |  | 0.3 | 0.5 | 0.19 | 0.81 | 0.7 |
| Vairaktaris 2009 | 0.43 | 0.45 | 0.12 | 0.88 | 0.57 |  | 0.29 | 0.41 | 0.3 | 0.7 | 0.71 |
| Vossen 2011 | 0.33 | 0.48 | 0.2 | 0.8 | 0.67 |  | 0.29 | 0.52 | 0.19 | 0.81 | 0.71 |
| Vossen 2011 | 0.32 | 0.46 | 0.22 | 0.78 | 0.68 |  | 0.29 | 0.52 | 0.19 | 0.81 | 0.71 |
| Weng 2011 | 0.25 | 0.54 | 0.21 | 0.79 | 0.75 |  | 0.25 | 0.53 | 0.22 | 0.78 | 0.75 |
| Weng 2010 | 0.26 | 0.57 | 0.17 | 0.83 | 0.74 |  | 0.25 | 0.53 | 0.22 | 0.78 | 0.75 |
| Woo 2007 | 0.36 | 0.45 | 0.18 | 0.82 | 0.64 |  | 0.36 | 0.45 | 0.19 | 0.81 | 0.64 |
| Turkmen 1997 | 0.27 | 0.59 | 0.14 | 0.86 | 0.73 |  | 0.43 | 0.35 | 0.22 | 0.78 | 0.57 |
