# Supplementary material for: PAI-1 4G/5G Polymorphism Contributes to Cancer Susceptibility: Evidence from Meta-Analysis
Source: PLoS One. 2013 Feb 20;8(2):e56797. doi: 10.1371/journal.pone.0056797 (PMC3577655; doi:10.1371/journal.pone.0056797)
Supplement: Table S2 — Stratification analyses of the I2 and 95%/97.5% confidence interval. If I2 = 0, the one-sided 97.5% CI is presented. Otherwise, a two-sided 95% CI is performed. (DOC) [file pone.0056797.s002.doc]

| **Table S2. Stratification analyses of the *I^2^* and 95% /97.5% confidence interval.** | | | | | | | | |  |  |  |
| --- | --- | --- | --- | --- | --- | --- | --- | --- | --- | --- | --- |
| **Variables** | **Sample size** | | | **4Gvs5G** | | **4G/4Gvs5G/5G** | | **4G/4Gvs4G/5G** | | **4G/4Gvs4G/5G+5G/5G** | |
|  | **n** | **case** | **control** | ***I^2^(%)*** | **95%/97.5%CI** | ***I^2^(%)*** | **95%/97.5%CI** | ***I^2^(%)*** | **95%/97.5%CI** | ***I^2^(%)*** | **95%/97.5%CI** |
| **Total** | 25 | 9205 | 11827 | 49.5 | 0.49-0.50 | 51.9 | 0.51-0.53 | 0 | 0-0.0002^a^ | 20.8 | 0.20-0.21 |
| **Tumor type** |  |  |  |  |  |  |  |  |  |  |  |
| Breast cancer | 8 | 4062 | 3320 | 48.3 | 0.47-0.49 | 48.8 | 0.48-0.50 | 6 | 0.05-0.07 | 22.8 | 0.22-0.24 |
| Colorectal cancer | 5 | 2426 | 4838 | 0 | 0-0.0005 ^a^ | 0 | 0-0.0005 ^a^ | 0 | 0-0.0005 ^a^ | 0 | 0-0.0005 ^a^ |
| Ovarian cancer | 2 | 794 | 912 | 0 | 0-0.002 ^a^ | 0 | 0-0.002 ^a^ | 55.1 | 0.53-0.57 | 22.9 | 0.21-0.25 |
| Endometrial cancer | 2 | 346 | 513 | 0 | 0-0.004 ^a^ | 0 | 0-0.004 ^a^ | 0 | 0-0.004 ^a^ | 0 | 0-0.004 ^a^ |
| Oral cancer | 2 | 357 | 450 | 87.3 | 0.85-0.90 | 86.5 | 0.84-0.89 | 0 | 0-0.005 ^a^ | 67.7 | 0.64-0.71 |
| Others | 6 | 1220 | 1794 | 57.8 | 0.56-0.60 | 63.2 | 0.61-0.65 | 0 | 0-0.001 ^a^ | 24.6 | 0.23-0.26 |
| **Ethnicity** |  |  |  |  |  |  |  |  |  |  |  |
| Caucasian | 17 | 6794 | 8424 | 56.8 | 0.56-0.58 | 59.6 | 0.59-0.60 | 3.6 | 0.03-0.04 | 25.3 | 0.25-0.26 |
| Asian | 6 | 1001 | 2036 | 45.9 | 0.44-0.48 | 44.8 | 0.43-0.47 | 17.3 | 0.16-0.19 | 37.8 | 0.36-0.40 |
| Mixed | 2 | 1410 | 1367 | 0 | 0-0.001 ^a^ | 0 | 0-0.001 ^a^ | 0 | 0-0.001 ^a^ | 0 | 0-0.001 ^a^ |
| **Control source** |  |  |  |  |  |  |  |  |  |  |  |
| Hospital based | 17 | 2013 | 3100 | 43 | 0.42-0.44 | 48.1 | 0.47-0.49 | 0 | 0-0.0007 ^a^ | 0 | 0-0.0007 ^a^ |
| Population based | 8 | 7192 | 8727 | 0 | 0-0.0002 ^a^ | 0 | 0-0.0002 ^a^ | 8.4 | 0.08-0.09 | 0 | 0-0.0002 ^a^ |
| **Sample size(both cases and controls)** | | | |  |  |  |  |  |  |  |  |
| <500 | 15 | 1554 | 2401 | 40 | 0.38-0.41 | 46.5 | 0.45-0.48 | 0 | 0-0.0009 ^a^ | 0 | 0-0.0009 ^a^ |
| ≥500 | 10 | 7651 | 9426 | 0 | 0-0.0002 ^a^ | 0 | 0-0.0002 ^a^ | 5 | 0.04-0.05 | 0 | 0-0.0002 ^a^ |

a: Because the *I^2^* is equal to 0, the CI is presented as one-sided, 97.5% confidence interval. The others which do not have a note of “a” represent a 95%CI.
